# AGE AND RACE PATTERNS IN TWO TYPES OF OBJECTIFICATION OF WOMEN

**DOI:** 10.1093/geroni/igae098.4112

**Published:** 2024-12-31

**Authors:** Aurora Sherman

**Affiliations:** Oregon State University, Corvallis, Oregon, United States

## Abstract

Objectification of women can take several forms, including denial of autonomy, sexualization, and self-objectification. Objectification is a dehumanizing experience with significant negative psychological and physical health consequences which has largely been studied in majority White and younger samples of women. This study surveyed 349 women aged 50 and older, who are Latina (n= 107), African American (n= 125), or White (n= 117). Objectification was measured by the Self-Objectification Beliefs and Behaviors Scale (SOBBS, Lindner & Tanleff-Dunn, 2017) and by the Invisibility subscale from the Self-Objectification Scale (SOS-I, Talmon & Ginsburg, 2016). The web-based survey was administered by Qualtrics Panel Services. Results indicate that the scales are reliable for all three groups and across mid-life and older women. Further, ANCOVAs with self-rated health as a covariate revealed no significant differences in either objectification measure based on race or ethnic group, suggesting that objectification is a shared experience for women from these three backgrounds. Finally, the ANCOVA also showed a significant age-group difference in the SOBBS scores (F(1, 324) = 5.18, p <.03) but not in Invisibility scores (F(1, 325) = 2.06, ns). Mid-life women reported higher self-objectification experiences (EMM = 42.62, SE = 1.33) compared to women older than 65 (EMM = 38.32, SE = 1.33). There were no significant interactions of race group with age group for either measure. Results are discussed in the context of Objectification and Life Course Theories.

